# Dual Urinary Tract Pathology in a Frail Elderly Man

**DOI:** 10.7759/cureus.108211

**Published:** 2026-05-04

**Authors:** Olufemi Pirisola

**Affiliations:** 1 Geriatrics, Royal Gwent Hospital, Newport, GBR

**Keywords:** complicated urinary tract infection, frail elderly, prostatic abscess, pyelonephritis, uti

## Abstract

A prostatic abscess is a rare but critical complication of urinary tract infections that may coexist with upper urinary tract involvement, such as pyelonephritis. This case report describes an 84-year-old frail gentleman with dementia who was admitted with a fall into the Accident and Emergency Unit. He was noted to be febrile, with suprapubic tenderness and raised infection markers. He underwent a CT trauma series, which revealed right kidney pyelonephritis with a prostatic abscess. A broad-spectrum intravenous antibiotic was started with no improvement, and the patient was not suitable for surgical drainage. He received multiple courses of long-term antibiotics, and his condition improved with the resolution of the abscess. Early imaging is generally indicated for patients with atypical presentations, poor response to therapy, or underlying immunocompromised status. In this case, it helped to confirm acute pyelonephritis and identify complications, including abscess formation.

## Introduction

Prostatic abscess is an uncommon but serious urological condition that occurs in 2.7% to 6% of men with acute bacterial prostatitis [1]. It is common among men in the fifth and sixth decades of life. The simultaneous occurrence of pyelonephritis and prostate abscess is a particularly challenging clinical scenario, requiring prompt recognition and aggressive management. This dual pathology typically affects immunocompromised patients, especially those with diabetes mellitus, though 23% occur in immunocompetent individuals. It is increasingly associated with multidrug-resistant organisms, recent urological procedures, and urinary tract abnormalities [1,2]. The simultaneous occurrence with pyelonephritis is exceptionally rare and poorly characterized in the literature. We present a case of pyelonephritis and prostatic abscess in an immunocompetent, frail older man, highlighting the diagnostic approach, management plan, and clinical outcomes. This case is significant because it challenges the immunocompromise paradigm by occurring in an immunocompetent, frail older man, highlights frailty as a potentially underrecognized risk factor, and addresses critical knowledge gaps regarding the diagnosis and management of dual upper- and lower-genitourinary suppurative infections.

## Case presentation

An 84-year-old gentleman, with a background of dementia, hypertension, and chronic obstructive pulmonary disease (COPD), was discovered after an unwitnessed fall at his residential care facility. He was found conscious on the floor but was unable to get up unaided, prompting ambulance transfer to the hospital. On examination, he had mild suprapubic tenderness, decreased breath sounds bilaterally with crackles at the right lung base, and was febrile, tachycardic, and tachypneic. His oxygen saturation was 90% while receiving 2 L of supplemental oxygen. Laboratory investigations demonstrated elevated inflammatory markers (Table 1). Empirical therapy was initiated while awaiting results from urine and blood cultures, which subsequently guided narrowing of the antimicrobial spectrum.

**Table 1:** Laboratory investigations on admission.

Parameter	Result	Reference range
White cell count	15.6 × 10⁹/L	4.0–11.0 × 10⁹/L
Neutrophils	14.2 × 10⁹/L	1.5–8.0 × 10⁹/L
C-reactive protein	248 mg/L	<10 mg/L
Creatinine	171 µmol/L	60–110 µmol/L

**Figure 1:**
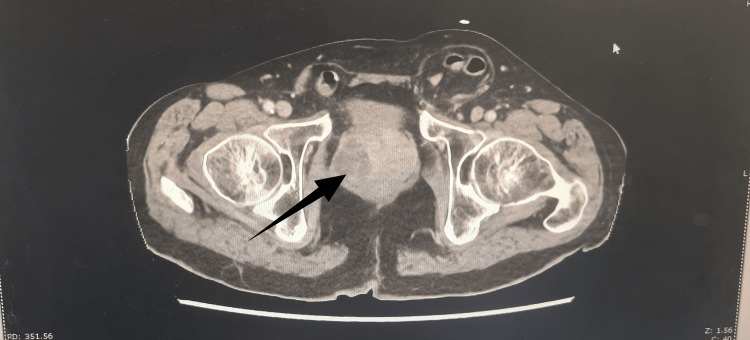
Contrast-enhanced CT scan of the pelvis in the axial view showing features suggestive of a prostrate abscess in the right lobe. The black arrow points to the abscess in the right lobe of the prostate.

**Figure 2:**
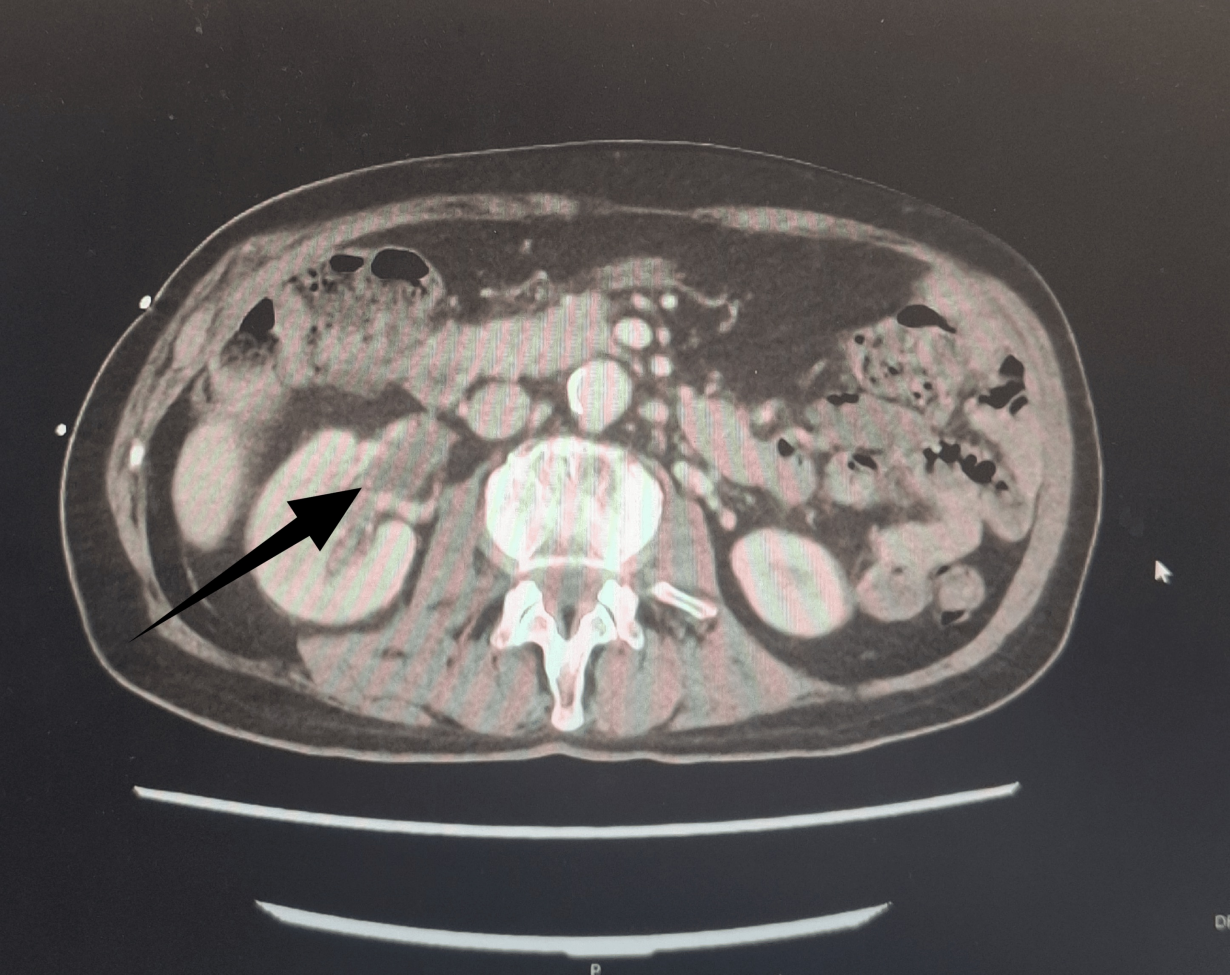
Contrast-enhanced CT scan of the abdomen in the axial view showing features suggestive of pyelonephritis. The black arrow points to the pathology on the CT scan.

After five days, the urine culture was positive for *Escherichia coli*, sensitive to ciprofloxacin. Imaging via a CT trauma series identified inflammatory changes affecting the right kidney and ureter, alongside the prostate, which was enlarged, containing a well-defined hypodense fluid collection in the right lobe measuring approximately 3.8 cm, consistent with a prostatic abscess (Figure 1). The right kidney showed areas of corticomedullary differentiation loss, consistent with pyelonephritis (Figure 2).

Empiric treatment was initiated with intravenous piperacillin-tazobactam, and the Urology team subsequently assessed the patient. Given his advanced age and multiple co-existing medical conditions, surgical management was considered inappropriate; therefore, a prolonged course of oral ciprofloxacin for a minimum of four weeks was recommended [2,3]. The patient began to respond to the antibiotic after 48 hours, as evidenced by the absence of fever and stable vital signs. He was discharged after three weeks of admission to be seen in the clinic after four weeks.

## Discussion

This case outlines the diagnostic challenges encountered when managing genitourinary infections in frail, elderly populations with multiple comorbidities, particularly those with cognitive impairment [4]. The American College of Radiology Appropriateness Criteria strongly advocate early imaging in complicated patients with suspected acute pyelonephritis, especially those of advanced age, suffering from diabetes, immunocompromised, or having no response to treatment [5]. Contrast-enhanced CT of the abdomen and pelvis is the imaging modality of choice. It has a higher detection rate for acute pyelonephritis at 84.4% compared to ultrasound at 40% [5,6]. The CT trauma series performed at presentation in the hospital in this case proved invaluable in diagnosing both pyelonephritis and prostatic abscess, which would have been difficult given that the patient could not reliably communicate symptoms due to his dementia [4-6].

Elderly patients with dementia represent a particularly vulnerable population for complicated urinary tract infections. Studies have shown that cognitive impairment, functional disability, and urinary incontinence are independent predictors of genitourinary infections in the oldest old [4]. The challenges of diagnosing infections in patients with advanced dementia are well-documented, as these patients often present with atypical symptoms and cannot provide reliable histories [4].

The successful conservative management of this patient’s dual pathology should be interpreted within the broader spectrum of prostatic abscess presentations, as cases arising after procedural manipulation, such as prostate biopsy, have been reported to require more invasive drainage strategies. This contrast underscores the importance of tailoring management to etiology, clinical context, and patient-specific risk factors [7]. While surgical drainage has traditionally been considered standard treatment for abscesses, accumulating evidence supports antibiotic therapy alone for selected cases. A comprehensive literature review reported an overall success rate of 85.9% for antimicrobial therapy without drainage in bacterial abscesses, with abscess size as the most important predictor of outcome [2]. Recent comparative studies show that transperineal drainage may result in shorter hospital stays and lower mortality compared to conservative management, particularly for large abscesses [8]. Studies show that renal abscesses measuring 5 cm or smaller can be fully resolved with intravenous antibiotics in some cases [9,10].

The choice of broad-spectrum antibiotics in this case aligns with current recommendations for complicated urinary tract infections in elderly patients. The inflamed prostate in acute infections is more permeable to antibiotics than in chronic disease, allowing better tissue penetration [1,3]. Current guidelines recommend a minimum of two weeks of antibiotics for acute bacterial prostatitis, with abscesses often requiring longer courses of four to six weeks [3]. The emergence of multidrug-resistant organisms, including extended-spectrum beta-lactamase-producing *Enterobacteriaceae* and methicillin-resistant *Staphylococcus aureus*, has complicated antibiotic selection, with studies showing 75% resistance to first-generation antibiotics [2]. *Staphylococcus aureus* has emerged as the most common pathogen in prostatic abscess, accounting for 56% of cases in recent series [2].

For concurrent pyelonephritis, evidence supports shorter antibiotic courses than traditionally prescribed. Recent meta-analyses demonstrate that five to seven days of fluoroquinolone therapy is as effective as 10-14 days for uncomplicated pyelonephritis [11,12]. However, in complicated cases with concurrent prostatic abscess, longer treatment duration is warranted.

This case illustrates the need for individualized treatment approaches in frail elderly patients. Although surgical drainage might offer faster resolution and shorter hospital stays in suitable candidates, prolonged antibiotic therapy can achieve comparable outcomes in patients with prohibitive surgical risk [2,3,7,13]. Risk factors for prostatic abscess include diabetes mellitus, advanced age, COPD, and recent urological instrumentation, all of which should be considered when determining management strategy [1].

## Conclusions

This case highlights the importance of maintaining a high index of suspicion for complicated genitourinary infections in frail older adults presenting with non-specific or atypical symptoms. The coexistence of pyelonephritis and prostatic abscess, although rare, can occur even in immunocompetent individuals and may be difficult to recognize in patients with cognitive impairment. Early imaging plays a crucial role in establishing the diagnosis and identifying associated complications, allowing timely and targeted management. While surgical drainage remains a standard approach for many prostatic abscesses, prolonged antibiotic therapy may provide an effective alternative in carefully selected patients who are poor surgical candidates. This case emphasizes the need for individualized treatment strategies, multidisciplinary involvement, and early radiological assessment to improve outcomes in complex urinary tract infections among frail elderly populations.
